# Editorial: Exploring obesity risk, prevention, and research innovation in the first 2000 days of life, volume II

**DOI:** 10.3389/fendo.2024.1372551

**Published:** 2024-02-05

**Authors:** Miaobing Zheng, Gengsheng He, Sarah Taki, Li Ming Wen

**Affiliations:** ^1^ Institute for Physical Activity and Nutrition, School of Exercise and Nutrition Sciences, Deakin University, Geelong, VIC, Australia; ^2^ School of Public Health, Fudan University, Shanghai, China; ^3^ Health Promotion Unit, Population Health Research and Evaluation Hub and Sydney Institute for Women, Children and Their Families, Sydney Local Health District, Sydney, NSW, Australia; ^4^ Sydney School of Public Health, Faculty of Medicine and Health and Centre of Research Excellence in the Early Prevention of Obesity in Childhood (EPOCH), The University of Sydney, Sydney, NSW, Australia

**Keywords:** first 2000 days, obesity prevention, childhood obesity, risk factors, cardiometabolic health

The high and rising prevalence of obesity is an intractable global health concern with far-reaching health, psychosocial and economic implications (1). Compelling evidence has revealed that obesity risk originates in early life and tracks across the life course with 39 million children under the age of 5 already being overweight or obese globally in 2020 (1, 2). The developmental origins of health and disease (DOHAD) theory also supports the importance of early life exposures in programming of health and disease including obesity and cardiometabolic health in later life (3). Obesity is a multifactorial disease attributable to a multitude of factors at individual, behavioural, and environmental levels, requiring urgent strategies to facilitate early prevention (4). The first 2000 days of life from conception to age five years have been identified as a critical and sensitive window for early obesity prevention and health promotion (5). Defining early life factors underpinning the development of obesity is imperative to inform the design of early obesity prevention interventions and strategies. However, our understanding of the early origins of obesity is limited. Volume II of our Research Topic showcases the latest research innovations exploring obesity-contributing factors in the first 2000 days and the impact of early obesity prevention interventions on later health outcomes.

The review by Cauzzo et al. summarised the findings of the latest studies investigating the connection between birth size, postnatal growth pattern, and cardiometabolic risk in childhood and adulthood with a focus on children born small for gestational age (SGA) or preterm. This review revealed that children with low birth weight, born SGA or preterm, and experienced postnatal rapid weight gain or catch-up growth are more likely to have adverse cardiometabolic outcomes including abnormal glucose-insulin metabolism, overweight and obesity, hypertension, endothelial dysfunction, dyslipidaemia, and metabolic syndrome. The review highlights the importance of supporting women of child-bearing age to deliver offspring with a normal birth size and implementing growth monitoring in the intrauterine and infancy periods to promote optimal cardiometabolic health across the lifespan.

In a large cohort of Chinese preschool children (n>2200), Jin et al. further demonstrated the vital contribution of the intrauterine metabolic environment in the development of childhood obesity. Several markers of lipid and insulin metabolism in the umbilical cord blood were found to be inversely associated with early childhood obesity outcomes. Receiver operating characteristic (ROC) curve analyses were conducted to identify the optimal cut-offs of cord metabolic factors for predicting obesity outcomes. This is the first study to report inverse associations between cord blood C-peptide and HbA1c levels and obesity outcomes at age 18 months. The study findings provide new evidence to support the crucial contribution of the intrauterine environment in programming later health.

Apart from intrauterine and postnatal growth indicators, behavioural factors such as physical activity also play a pivotal role in the development of obesity and associated health outcomes (4, 5). Eichner-Seitz et al. conducted a review to synthesize physical activity intervention strategies in infancy and early childhood and their impact on obesity prevention, cardiometabolic and bone health. Common intervention strategies involve the promotion of tummy time during infancy and muscle strength and motor development in early childhood, as well as parental and environmental interventions to increase child physical activity across early childhood, which have shown modest intervention effects. Considering the limitations of the current literature, the review proposed practical recommendations and novel strategies for researchers and public health professionals to promote physical activity from early childhood.

Dietary intake is another well-recognized obesity contributing factor (4, 5). Emerging evidence suggests dietary habits are developed early in life and may be tracked across the life course (6). Park et al. examined the intake trends of various food groups and nutrients during breakfast among Australian preschool children over three time points when children were aged 1.5, 3.5, and 5 years. Key food groups consumed during breakfast include grains, milk and alternatives, and discretionary items high in saturated fat and sodium, whereas vegetables were rarely consumed during breakfast. Low to moderate tracking of most food groups and nutrients was found over time. This study supports the initiation of dietary interventions from early life and provides valuable evidence to inform the design of interventions and guidelines to promote healthy breakfast.

Taken together, findings from this Research Topic underline the importance of early life in establishing a healthy trajectory across the life span. However, further rigorously designed research is needed to fully understand the causal underpinnings of childhood obesity to facilitate early obesity prevention.

## Author contributions

MZ: Writing – original draft, Writing – review & editing. GH: Writing – review & editing. ST: Writing – review & editing. LW: Writing – review & editing.
